# The Dynamic Landscape of the Coagulome of Metastatic Malignant Melanoma

**DOI:** 10.3390/ijms26041435

**Published:** 2025-02-08

**Authors:** Jean-Philippe Arnault, Kimberley Chemmama, Khedidja Ferroudj, Julien Demagny, Laurence Panicot-Dubois, Antoine Galmiche, Zuzana Saidak

**Affiliations:** 1 Service de Dermatologie, CHU Amiens, 80054 Amiens, France; arnault.jean-philippe@chu-amiens.fr; 2 UR7516 CHIMERE, UFR de Médecine, Université de Picardie Jules Verne, 80054 Amiens, France; ferroudjkhedidja@gmail.com (K.F.); saidak.zuzana@chu-amiens.fr (Z.S.); 3 Service de Biochimie, Centre de Biologie Humaine, CHU Amiens, 80054 Amiens, France; chemmama.kimberley@chu-amiens.fr; 4 Service d’Hématologie Biologique, Centre de Biologie Humaine, CHU Amiens, 80054 Amiens, France; demagny.julien@chu-amiens.fr; 5 Aix-Marseille Univ, INSERM 1263, INRAE 1062, C2VN, 13385 Marseille, France; laurence.panicot-dubois@univ-amu.fr

**Keywords:** tumor coagulome, metastasis, skin cutaneous malignant melanoma, cancer-associated thrombosis

## Abstract

The local expression of coagulation-related genes defines the tumor coagulome. The tumor coagulome plays a pivotal role in cancer-associated thrombosis (CAT) and hemostatic complications, such as venous thromboembolism (VTE), which are frequent in patients with advanced/metastatic cancer. Genomic analyses of human tumors, such as skin cutaneous melanoma (SKCM), have unveiled the complexity of the metastatic trajectories. However, no study to date has focused on the metastatic coagulome along these trajectories. Using bulk-tumor and single-cell analyses of primary SKCM, metastastic samples and circulating tumor cells (CTCs), we explored the coagulome of SKCM along metastatic progression. We identified consistent changes in the coagulome of SKCM metastases compared to primary tumors and observed metastatic site specificity. Compared to other metastatic sites, lung metastases of SKCM had a specific coagulome with a higher expression of *F3*, encoding Tissue Factor. Single-cell analyses were used to chart the inter- and intra-tumor heterogeneity and characterize the metastatic coagulome of SKCM. We found that a subpopulation of CTCs from SKCM expressed high levels of platelet genes, suggesting the contribution of CTC–platelet interactions to the CTC coagulome. These findings highlight the dynamic properties of the metastatic coagulome and its link to cancer progression.

## 1. Introduction

The coagulation cascade is a central actor in the process of cancer-associated thrombosis (CAT). CAT accounts for venous thromboembolism (VTE) that encompasses deep vein thrombosis (DVT) and its severe complications, such as pulmonary embolism (PE). The complications of VTE are frequently encountered in cancer patients and represent a significant source of mortality and morbidity [1]. Interestingly, the proteases of the coagulation cascade also potentially contribute to tumor progression, especially during metastasis, as suggested in various studies and different experimental settings [2]. During hematogenous tumor dissemination, circulating tumor cells (CTCs) might activate the coagulation cascade and form stable blood clots of cancer cells clustered with blood cells, such as platelets and red blood cells [3]. By doing so, the coagulation cascade might install optimal physical and biological conditions for cancer cell survival in the blood, vessel arrest and the initial steps of metastatic implantation in target organs [3,4,5]. The activation of the coagulation cascade is, however, not limited to the lumen of tumor vessels. Specific components of the coagulation cascade, together with blood cells, such as platelets within the tumor interstitial space, are emerging as key determinants of the tumor microenvironment (TME) [6,7,8]. Through their ability to interact with specific receptors on the surface of different cell types within the tumor, activated thrombin/factor IIa or factor Xa regulate the cellular interactions between cancer cells, platelets, red blood cells and myeloid cells within the TME [3,6,7]. Accordingly, the coagulation cascade is emerging as a determinant of tumor progression and response to medical treatment [9,10].

Skin cutaneous melanoma (SKCM) is an aggressive tumor type able to form distant metastases in lungs, bones and other target organs [11,12]. Compared to other types of primary tumors, SKCM patients have an average risk of VTE and PE. However, VTE and PE become more prevalent in late stages of SKCM, and CAT therefore represents a significant cause of mortality and morbidity in these patients [13,14,15,16,17]. Beyond thromboembolic complications, the coagulation cascade also potentially contributes to SKCM progression. Animal models based on intravenous injection of cancer cells suggest that an activated coagulation cascade may promote SKCM metastasis [18,19,20,21]. In patients with advanced SKCM treated with immune checkpoint blockers (ICBs), elevated markers of coagulation are associated with poor prognosis [22]. Reciprocally, the use of anticoagulants targeting factor Xa might be associated with a longer overall survival and progression free survival [23]. These observations collectively point to the importance of coagulation as a key biological determinant involved in vascular complications, tumor progression and response of SKCM to therapeutics.

Cancer metastasis is a complex multistep process [24]. Tumor genomic studies of SKCM metastasis show the coexistence of multiple evolutionary pathways and cancer trajectories in each patient [25,26]. Genomics and transcriptomics also enable system analyses of the coagulation-related genes (CRGs), i.e., the tumor coagulome [27]. These approaches have shown the existence of key differences in CRG expression between different types of primary tumors [27]. Among the genes that are heterogeneously expressed in human tumors and in SKCM, *F3* encodes Tissue Factor (TF), a key upstream regulator of the coagulation cascade, whose expression correlates with the risk of VTE [27]. In SKCM, the expression of CRGs has been correlated with basic tumor staging and found to be of potential prognostic value [28]. While these findings open the interesting possibility that the coagulome of SKCM might evolve along tumor progression, no study to date has yet examined the coagulome of metastatic SKCM. As single-cell RNA sequencing data become available for multiple types of metastases of SKCM, such as nodal metastases [29,30] and CTCs from SKCM patients [31], high-resolution studies of the metastatic coagulome of SKCM are now possible [32]. The present study aimed to explore the coagulome of metastatic SKCM and address its potential heterogeneity and dynamic regulation along tumor progression.

## 2. Results

### 2.1. The Coagulome of Metastases Compared to Primary Tumors

**Figure 1:**
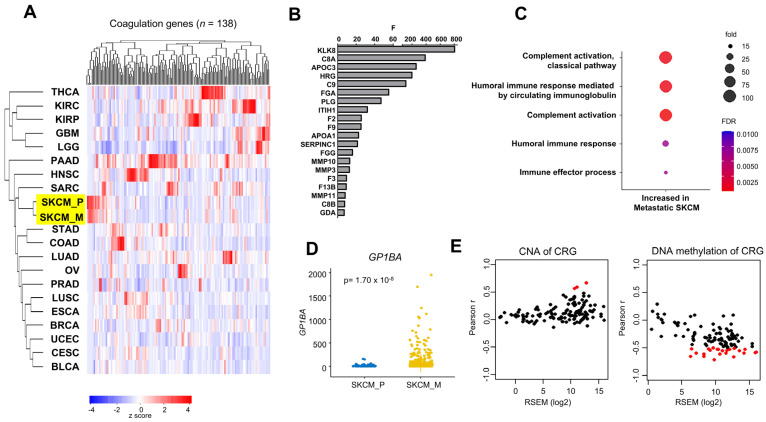
**Comparison of the coagulome of primary vs. metastastic SKCM.** (**A**) Hierarchical clustering based on the normalized expression levels of 138 CRGs. Primary SKCM tumors (SKCM_P) and metastases (SKCM_M), highlighted in yellow, and 19 other types of primary tumors from TCGA were analyzed. (**B**) The top 20 CRGs with higher variance in primary vs. metastatic SKCM (Fisher F test). (**C**) GO term analysis (GO Biological Process complete) of CRGs with at least a 2-fold higher expression in SKCM_M vs. SKCM_P (*n* = 16, *p* < 0.05, Student *t* test, FDR). (**D**) Violin plots showing the expression of *GP1BA*, the most differentially expressed gene, between SKCM_P and SKCM_M. (**E**) Plots showing the relationship between the expression of CRGs (RSEM) and their correlation with the copy number (CNA) or methylation levels (Pearson R). Points shown in red have an absolute Pearson correlation coefficient greater than 0.5. Points shown in black have an absolute Pearson r less than 0.5.

In order to examine the degree of conservation of CRG expression between primary tumor samples and metastases, we compared bulk RNA-seq from SKCM metastatic samples with 20 types of primary tumors (including primary SKCM), using CRG expression (z score values of RNA-seq by expectation maximization (RSEM) expression), from The Cancer Genome Atlas (TCGA) [33] (Figure 1A).

Hierarchical clustering based on the 138 CRGs from the hallmark “Coagulation” is shown in Figure 1A. While each primary tumor type has a specific CRG expression profile, SKCM metastases clustered with SKCM primary tumors and were distant from other primary tumors. This suggested that the tumor coagulome remain globally stable during SKCM progression. We turned our attention to the specific changes in gene expression found in metastatic samples. For each CRG, we performed an analysis of variance in primary vs. metastatic samples. CRGs were ranked according to their value of F, i.e., the ratio of variance in primary vs. metastatic samples (Figure 1B). A high F ratio indicates a decrease in variance in metastases compared to primary tumors, possibly reflecting a process of selection. Out of the 20 CRGs whose variance significantly differed between primary and metastastic SKCM samples, *F3* had an almost 10-fold reduction in variance in metastases compared to primary SKCM (9.5-fold decrease, *p* = 1.44 × 10^−56^) (Figure 1B). In addition to the analysis of variance, any significant changes in gene expression may also reflect the contribution of CRGs to SKCM metastasis. We compared the expression levels of each CRG in metastases and primary SKCM. We identified 16 CRGs with more than twofold higher average expression in metastases compared to primary SKCM (Figure S1, Table S1). A gene ontology (GO) analysis identified the terms “complement activation” (GO0006856) and “humoral immune response” (GO0002455) as being more represented in SKCM metastases than primary tumors (Figure 1C). Interestingly, the gene GP1BA, encoding Glycoprotein Ib Platelet Subunit Alpha, a receptor for von Willebrand factor (VWF), was the most differentially expressed in primary vs. metastatic SKCM (a 4.65-fold higher expression in metastases of SKCM compared to primary tumors, *p* = 0.0005) (Figure 1D). We carried out a stromal/immune analysis comparing metastases of SKCM stratified according to their GP1BA expression (high/low by median), which showed that the expression of this gene is linked to a more stromal (*p* = 9.70 × 10^−7^) and inflammatory/immune-infiltrated (*p* = 2.25 × 10^−24^) tumor microenvironment (Figure S2A). A separate Cibersortx analysis confirmed the greater absolute number of inflammatory/immune cells in SKCM metastases with high *GP1BA* (Figure S2B). We concluded that metastases of SKCM consistently display a more proinflammatory coagulome.

We explored the contribution of genomic events to the regulation of the metastatic coagulome by calculating a Pearson correlation coefficient between the expression level of each CRG and their copy number alterations (CNAs) in SKCM metastases (Figure 1E). Only three CRGs displayed a positive correlation (r > 0.5) between gene expression and CNA, suggesting a minor contribution of gene amplification/loss to the regulation of the metastatic coagulome of SKCM. Meanwhile, DNA methylation, an essential epigenetic mark, was negatively correlated (R < −0.5) with the expression of n = 23 CRGs in SKCM metastases (Figure 1E). We concluded that epigenetics plays a more prominent role in shaping the metastatic coagulome of SKCM than CNA.

### 2.2. Target Organ Specificity of the Metastatic Coagulome

**Figure 2:**
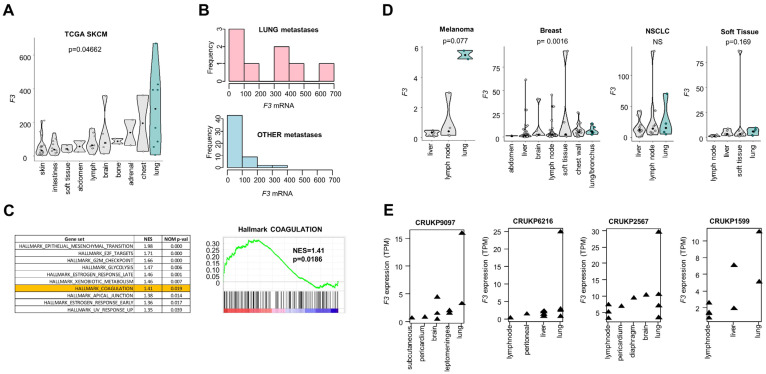
**Lung metastases of SKCM express higher F3 levels.** (**A**) *F3* expression analysis in different metastatic locations of SKCM from TCGA, *p* < 0.05 with Kruskal–Wallis test. Lung metastases are shown in turquoise, other metastatic sites in grey. (**B**) Distribution of *F3* expression in SKCM lung metastases (*n* = 8, pink) compared to other locations (*n* = 63, blue). (**C**) GSEA showing a significant enrichment in the hallmark “Coagulation” in lung metastases of SKCM compared to other metastatic sites. (**D**) *F3* expression analysis in metastastic samples from four types of tumors from the BCSGC cohort (*n* = 438). Note that *F3* levels are presented for metastastic locations with >2 samples. (**E**) *F3* expression in multiple matched samples from 4 patients with SKCM (PEACE study).

We examined CRG expression according to the metastatic locations of SKCM. Hierarchical clustering suggested that lung metastases of SKCM have a specific coagulome characterized by the strong expression of a cluster of 9 genes, including *F3* (Figure S3). Interestingly, lung metastases expressed higher median F3 levels than other metastatic locations in SKCM-TCGA (280.1 vs. 57.6 RSEM, i.e., 4.9-fold higher *F3* expression in lung metastases, *p* = 0.0466, Kruskal–Wallis) (Figure 2A).

In contrast to all other metastatic locations of SKCM analyzed, high expression of *F3* was found in 50% of lung metastases of SKCM (Figure 2B). In order to perform a systematic comparison of the entire transcriptome of lung SKCM metastases vs. other SKCM metastases, we used Gene Set Enrichment Analysis (GSEA). We confirmed the statistically significant enrichment of the hallmark “Coagulation” in lung metastases of SKCM (Normalized Enrichment Score (NES) = 1.41, *p* = 0.0186) (Figure 2C), with 53 CRGs accounting for the enrichment signal (Table S2). Among these genes, we noted, in addition to *F3,* other coagulation factors (*F2*, *F13B*, *F10*, *F11*), *VWF* (Von Willebrandt Factor) and *THBS1* (Thrombospondin 1). We concluded that lung metastases of SKCM display a specific coagulome with a frequent overexpression of *F3*.

We examined which pathological characteristics might be related to the high expression of *F3* found in lung metastases of SKCM. The high expression of *F3* was not related to CNA, whereas significantly lower levels of DNA methylation of the *F3* locus were noted (Figure S4). We applied the stromal/immune analyses to lung metastases of SKCM (n = 8) stratified according to *F3* expression. *F3*^high^ SKCM metastases had strikingly higher expression of stromal genes (Figure S5A). The detailed gene analysis presented in Table S3 indicates, for example, that the expression of the *FAP* gene (encoding Fibroblast Activation Protein Alpha) was more than 51 times higher in *F3*^high^ compared to *F3*^low^ metastases (Figure S5B). Immune score did not significantly differ between the two groups (*p* = 0.2445) (Figure S5C). Cibersortx analysis of lung metastases of SKCM did not find any significant association between *F3* and the immune cell composition either, analyzed as relative fractions or absolute scores (Figure S5D; Table S4). The findings suggested that the high expression of *F3* noted in lung metastases of SKCM was more closely related to the stromal characteristics of the metastatic tissue than to its infiltration by immune/inflammatory cells.

Independent validation of the association between high *F3* expression and lung location of metastases was obtained from two independent studies with bulk RNA-seq data from metastases of SKCM (Figure 2D,E). In the BCGSC cohort (British Colombia Genomics Sciences Center, POG570) [34], we confirmed significantly higher expression of *F3* in lung metastases of SKCM compared to all other metastatic locations of SKCM (*p* = 0.02, Figure 2D). Interestingly, lung metastases of breast carcinoma, non-small cell lung carcinoma, sarcoma/soft tissue tumors also ranked highest in *F3* expression (Figure 2D). The transcriptomic characterization of multiple metastatic samples in posthumous SKCM patients PEACE study [25] offered the advantage of comparing multiple matched samples from a small number of patients. As shown in Figure 2E, in four SKCM patients with 38 samples analyzed, lung metastases systematically ranked as the highest expressors of *F3* compared to other patient-matched metastatic locations (Figure 2E).

### 2.3. The Metastatic Coagulome at a Single-Cell Resolution

**Figure 3:**
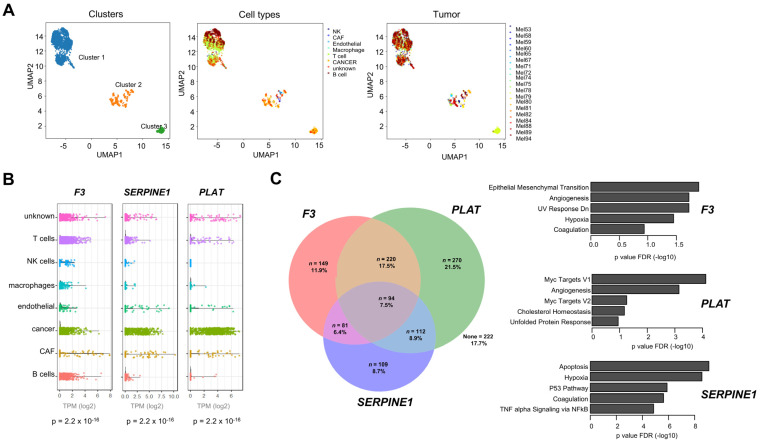
**Single-cell RNA seq analysis of the coagulome of metastatic SKCM.** (**A**) UMAP analysis of *n* = 4645 cells from 19 SKCM metastases (GSE72056), based on the expression levels of *n* = 138 CRGs. Note that cluster #1 includes all immune cells (NK cells, B, T cells and macrophages), while cancer cells, CAFs and endothelial cells from different patients appear in clusters #2 and #3. (**B**) Cell type-specific expression of the “core coagulome” genes in SKCM metastases. (**C**) A Venn diagram showing the overlap of *F3*, *PLAT*, and *SERPINE1* mRNA expression in cancer cells from GSE72056. The % of overlap is indicated in each case. The GO terms (MSigDB Hallmark) were identified using the 40 most correlated genes with each of the three genes of interest (Pearson r).

In order to characterize the metastatic coagulome of SKCM at the single-cell level, we analyzed scRNA-seq data from *n* = 4645 cells from 19 metastases of SKCM (GSE72056) [29], with gene expression given as log2 of TPM (transcripts per million). Dimension reduction analysis with UMAP based on the expression of the 138 CRGs unveiled three clusters: cluster #1 included immune cells, while clusters #2 and #3 consisted primarily of cancer cells, cancer-associated fibroblasts (CAFs) and endothelial cells (Figure 3A).

Table S5 shows the list of genes significantly enriched in each of the main cellular populations found in SKCM metastases. We noted the dominant contribution of cancer cells to the expression of the “core coagulome”, i.e., the CRGs directly upstream of thrombin/plasmin activation (*F3*, *PLAT* and *SERPINE1*) (Figure 3B). We found no evidence of simultaneous mRNA expression of *F3*, *SERPINE1* or *PLAT*, with only a small fraction of cancer cells expressing all three genes (7.5%) (Figure 3C). We examined the context in which SKCM cells express these genes by performing a GO analysis on the most correlated genes for each gene of interest (Figure 3C, Table S6). This analysis pointed to “Epithelial/Mesenchymal Transition” in SKCM cells expressing *F3*. Conversely, *PLAT* expression correlated to “MYC signaling” and *SERPINE1* was linked to “Apoptosis”. The expression of these three genes varied greatly between patient and within each tumor (Figure S6A). In general, inter-patient and intra-tumor variance of CRG expression were positively correlated (r = 0.68) (Figure S6B). Interestingly, however, this analysis identified *PLAT* expression as an outlier with higher intra-tumor than inter-tumor heterogeneity (Figure S6B). Compared to cancer cells, non-malignant cells had different cellular coagulomes. T and B cells infiltrating SKCM metastases expressed in general low levels of most CRGs, while CAFs were characterized by high expression of CRGs related to complement (*C3*, *C1Q*, *C1R*) (Figure S7A). The expression of complement genes was strongly correlated with “TNF-alpha signaling via NFKB”, suggesting a key regulatory role of inflammation in this setting (Figure S7B).

### 2.4. The Coagulome of Circulating Tumor Cells (CTCs)

**Figure 4:**
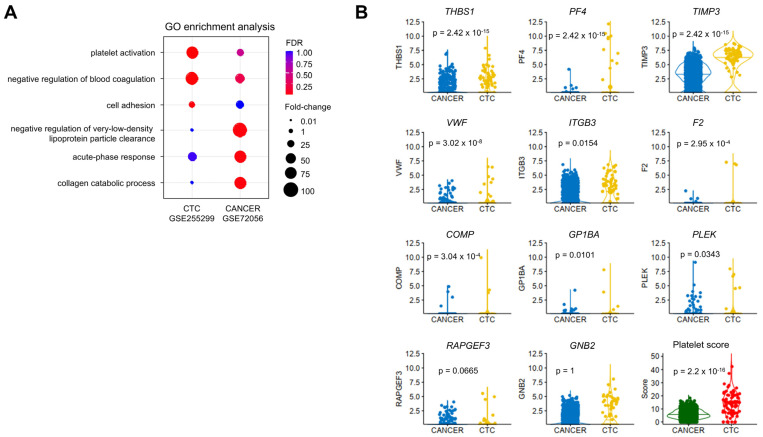
**The coagulome of circulating tumor cells (CTCs) from SKCM.** (**A**) GO term enrichment analysis of CTCs (GSE255299) compared to cancer cells from GSE72056, using the most significantly differentially expressed genes, between the two groups. (**B**) Violin plots showing the most differentially expressed platelet-related genes between CTCs and cancer cells. The overall “Platelet score” is obtained from the combination of the corresponding 11 platelet genes.

In order to investigate the coagulome of CTCs from SKCM, we used scRNA-seq data from *n* = 75 CTCs analyzed from 7 SKCM patients [31] (GSE255299). Raw data from GSE255299 were processed and analyzed according to Tirosh et al. [29] for further comparison. Gene expression data from GSE255299 were then merged with cancer cell data from GSE72056. Upon UMAP analysis, we noted overlapping clusters between the cells from the two studies, overall indicating comparability of the gene expression profiles in the two studies (Figure S8). A direct comparison indicated that 32 CRGs were expressed at significantly higher levels in CTCs compared to cancer cells from solid metastases (Table S7). Strikingly, the corresponding CRGs were enriched in genes related to platelet function, as confirmed by GO term analysis: a 61.7-fold enrichment for the GO term “Platelet activation” (*p* = 8.96 × 10^−7^) (Figure 4A).

**Figure 5:**
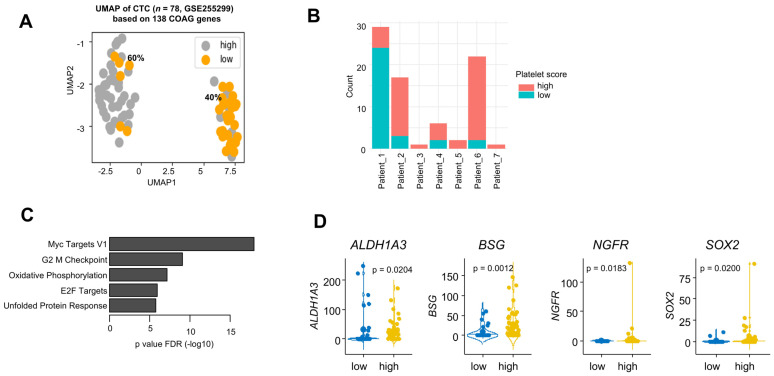
**A subset of CTCs with a platelet coagulome.** (**A**) UMAP analysis of CTCs (GSE255299) according to their expression of CRGs. The colors indicate the platelet score (orange = low, grey = high). (**B**) Individual patient analysis (*n* = 7 patients) of CTCs from GSE255299. Note that CTCs with a positive platelet score are in pink and platelet-negative CTCs are in blue. (**C**) GO term enrichment analysis of genes most correlated with the platelet score (Spearman) in CTCs. (**D**) Expression analysis of four stemness markers according to the platelet score in CTCs of SKCM.

The expression levels of 11 genes (*THBS1*, *PF4*, *TIMP3*, *VWF*, *ITGB3, F2*, *COMP*, *GP1BA*, *PLEK*, *RAPGEF3* and *GNB2*, later referred to as the “platelet signature”) were significantly higher in CTCs compared to cancer cells from “solid” metastases (Figure 4B). Conversely, *F3* and *SERPINE1* were expressed at lower levels in CTCs, and we found no significant change in the expression levels of *PLAT* in CTCs (Figure S9). A UMAP analysis suggested the existence of two clusters, representing 60% and 40% of CTCs (Figure 5A).

Importantly, the distribution of CTCs into two clusters overlapped with the platelet signature defined earlier (Figure 5A). An individual patient analysis showed that the proportion of CTCs expressing platelet genes varied from 17.2% to 100% according to the patient (Figure 5B). Finally, we addressed the functional state of CTCs with a high platelet signature: a GO term analysis applied to the list of genes whose expression levels positively correlated with the platelet signature identified “MYC TARGETS v1” as highly enriched (*p* = 9.88 × 10^−8^) (Table S8, Figure 5C). Interestingly, four genes known to be related to cancer cell stemness in malignant melanoma (*ALDH1A3*, *BSG*, *NGFR*, *SOX2*) [35] were expressed at significantly high levels in CTCs with a high “platelet signature” (*p* = 0.0204, 0.0012, 0.0183, 0.0200, respectively).

## 3. Discussion

Despite the fact that the risk of VTE is the highest in advanced stages of cancer, the metastatic coagulome remains poorly characterized [1,6]. Here, we show that metastases of SKCM maintain the overall characteristics of the primary tumor coagulome. Metastases nevertheless display specific changes with a globally more inflammatory coagulome. We also observed consistent changes in the coagulome of different metastatic sites: *F3*, encoding TF, the master regulator of cancer-associated thrombosis, was expressed at higher levels in lung metastases compared to other metastatic sites, in TCGA and two independent cohorts of SKCM. We examined the coagulome of metastatic SKCM at a single-cell resolution. In addition to showing the specific contribution of different cell types to the bulk tumor coagulome, this analysis suggested the contribution of inflammatory and oncogenic signaling to the regulation of CAT in SKCM. Finally, we observed that CTCs frequently overexpress CRGs related to platelet function. At this stage, our study has a few important limitations. The first limitation is inherent to the correlative nature of our study, preventing us from drawing mechanistic conclusions regarding the role of the tumor coagulome in metastatic progression. Clearly, experimental studies are required to show the active contribution of coagulation in SKCM dissemination. A second limitation is the small number of samples and patients that were available, especially for single-cell analyses, along with the heterogeneous and fragmentary reporting of clinical data. More studies with larger and better-annotated cohorts of cancer patients are required to derive prognostic/predictive information from gene expression signatures based on the metastatic tumor coagulome. Along the same line, our study did not address the regulation of the cancer coagulome by medical treatments used in SKCM patients due to fragmentary availability of the corresponding data. Considering that BRAF kinase inhibitors or ICBs could directly regulate the coagulome of cancer cells, further studies testing the effects of medical treatments in patients with metastatic SKCM are required [16,17,36]. A final and important limitation of our study was its restriction to gene expression analyses. A proteomic characterization of the metastatic coagulome and the analysis of functional coagulation assays would have been valuable, but the corresponding information is currently not available. Despite these limitations, our study represents, to the best of our knowledge, the first study that directly addresses the evolution of the tumor coagulome along cancer progression and metastasis. Overall, our findings support the idea of a dynamic tumor coagulome, with specific changes in CRG expression in advanced/metastatic stages.

The high expression of *F3* that we noted in lung metastases of SKCM is an interesting observation that has, to our knowledge, not yet been reported. Importantly, the transcriptional landscape of cancer metastases should be considered as the confluence of the transcriptional program of the cancer itself with that of the target tissue [37]. From our observations, it is not possible to determine whether the high expression of *F3* found in lung metastases is a characteristic conferred by the lung tissue or an inherent trait of the metastasis itself. Whether a procoagulant phenotype associated with high *F3* expression, promotes the growth of lung metastases therefore remains speculative. Importantly, however, experimental animal models based on intravenous injection of cancer cells suggest a possible contribution of *F3* to lung metastases of SKCM [20,21] and other types of tumors with a strong metastatic lung tropism [38]. Recent comprehensive reviews on metastasis organotropism do not mention the contribution of coagulation [39], but it is tempting to speculate that lung metastases of SKCM could mimic the process of pulmonary embolism. A recent study put emphasis on physical constraints found in the circulation as a determinant of metastatic tropism [40]. An active coagulation cascade might efficiently promote blood clotting in conditions of low blood flow found in the venous circulation [41], favoring SKCM arrest in the lung. The possibility that a cancer procoagulant program contributes to metastatic lung tropism of SKCM deserves further exploration.

Our study suggests the existence of a subpopulation of CTCs with characteristics of a “platelet coagulome” in SKCM patients. Whether this observation reflects true CTC–platelet adhesion or other phenomena, for example, vesicle transfer, remains unknown. However, a number of recent studies directly point to stable physical interactions established between CTCs and host platelets in tumors other than SKCM [42,43,44,45,46]. A growing body of research supports the role of cellular clustering and cellular cooperations as crucial events to support cancer metastasis [3]. Compared to single CTCs analyzed in the blood of breast cancer patients, clusters of CTCs are characterized by a specific tumor biology dominated by increased expression of cancer stemness genes [47,48]. The significant enrichment in MYC target genes and the high expression of cancer stemness genes that we observed in CTCs with a platelet gene signature suggest the pro-oncogenic nature of this interaction, in agreement with the findings of studies in other tumor types [42]. The regulation of CTC–platelet interactions in SKCM deserves further investigation.

Metastasis is a complex and multistep process that represents a major challenge in cancer treatment [24,25,26]. From our analysis of SKCM emerges the concept of a dynamic tumor coagulome, with different trajectories depending on the nature of the metastatic sample or cell type examined. Our findings also shed light on the variety of cellular events that may regulate the tumor coagulome. In addition to genomic instability, epigenetics and transcriptional regulation, cellular interactions with platelets may modulate the cancer coagulome. This apparent complexity and the heterogeneity of the metastatic coagulome obviously represent a hurdle in applying basic science to the prevention of VTE in cancer patients [1]. Further studies are required to precisely define the extent of the mosaic tumor coagulome within individual patients with advanced/metastatic cancers.

## 4. Materials and Methods

### 4.1. Cohorts

Data from the TCGA-SKCM cohort [33] and 19 other TCGA cohorts as well as from the Pan-cancer Analysis of Advanced and Metastatic Tumors cohort from BCSGC (British Colombia Genome Sciences Centre) [34] were retrieved through https://cbioportal.org on 5 January 2024 [49,50]. Transcriptomic data from metastatic SKCM samples from the Posthumous Evaluation of Advanced Cancer environment (PEACE) study [25] were retrieved from Zenodo data repository https://zenodo.org/records/7673904 on 15 August 2024. Further details can be found in the Supplementary Materials and Methods.

### 4.2. Single-Cell Melanoma Analyses

Single-cell transcriptomic data from metastatic SKCM samples were retrieved from GSE72056 [29] and GSE255299 [31] via the Gene Expression Omnibus (GEO). More detail is provided in the Supplementary Materials and Methods section.

### 4.3. Gene Set Enrichment Analysis (GSEA), Gene Ontology (GO) Analysis

GSEA was performed using desktop application https://www.gsea-msigdb.org/gsea/index.jsp on 15 November 2024 using version 4.3.2 and Hallmark gene sets, as detailed in Supplementary Materials and Methods. Gene Ontology (GO) analyses were carried out using Enrichr [51] or PANTHER (Protein ANalysis THrough Evolutionary Relationships, http://www.pantherdb.org/) [52].

### 4.4. Tumor Microenvironment Analysis

Stromal/immune scores were used to evaluate the infiltration levels of stromal and immune cells based on gene expression data [53]. Cibersortx (https://cibersortx.stanford.edu) was used to quantify the levels of 22 cell subsets using leukocyte gene signature matrix LM22 [54,55].

### 4.5. Dimensionality Reduction Methods

The dimensionality reduction method UMAP (Uniform Manifold Approximation and Projection) was implemented in Python language (Spider v5) with parameter settings: n_components = 2, n_neighbors = 100, min_dist = 0.0, random_state = 42.

### 4.6. Statistics

Statistical analyses were performed either with R version 4.3.1 (https://www.r-project.org) or Python (Anaconda/spider 5.4.3). Groups were compared using non-parametric tests, either Wilcoxon–Mann–Whitney for comparisons of two groups or Kruskal–Wallis for more than two groups, unless otherwise stated. False discovery rate (FDR) correction was applied when appropriate. Heatmaps were created using the R library gplots (version 3.1.3.1), using Ward.D2 clustering. Correlation analyses were conducted using R package Hmisc (version 5.1-2). UMAP analyses were carried out using Python libraries Pandas, NumPy and umap.

## Data Availability

The data presented in this study are available from Gene Expression Omnibus (GEO) at https://www.ncbi.nlm.nih.gov/gds, reference numbers GSE72056 and GSE255299, accessed on 5 January 2024. TCGA and BCSGC data are available through cBioportal (https://www.cbioportal.org/), accessed on 5 January 2024. Data from the PEACE study are available from Zenodo (https://doi.org/10.5281/zenodo.7673904), accessed on 1 February 2024. R and Python codes used in this study are available from the authors upon request.

## Supplementary Materials

The following supporting information can be downloaded at https://www.mdpi.com/article/10.3390/ijms26041435/s1.

## Abbreviations

The following abbreviations are used in this manuscript:

CAF	Cancer-Associated Fibroblast
CAT	Cancer-Associated Thrombosis
CNA	Copy Number Alteration
CRG	Coagulation-Related Gene
CTC	Circulating Tumor Cell
FAP	Fibroblast Activation Protein
GEO	Gene Expression Omnibus
GO	Gene Ontology
GSEA	Gene Set Enrichment Analysis
GP1BA	Glycoprotein Ib Platelet Subunit Alpha
ICB	Immune Checkpoint Blocker
NES	Normalized Enrichment Score
PE	Pulmonary Embolism
RSEM	RNA-Seq by Expectation Maximization
SKCM	Skin Cutaneous Melanoma
TCGA	The Cancer Genome Atlas
TF	Tissue Factor
THBS1	Thrombospondin 1
TME	Tumor Microenvironment
TPM	Transcripts Per Million
UMAP	Uniform Manifold Approximation and Projection
VTE	Venous Thromboembolism
VWF	Von Willebrand Factor
