# Supplementary material for: The Dynamic Landscape of the Coagulome of Metastatic Malignant Melanoma
Source: Int J Mol Sci. 2025 Feb 8;26(4):1435. doi: 10.3390/ijms26041435 (PMC11855523; doi:10.3390/ijms26041435)
Supplement: Supplementary file 1 [file ijms-26-01435-s001.zip › Supplementary Figures.pdf]

Supplementary figures to J-P. Arnault et al.

The dynamic landscape of the coagulome of metastatic malignant melanoma

Figure S1. Differential expression analysis of CRG in primary vs metastatic SKCM-TCGA. A volcano plot showing differentially expressed CRG (log2 fold-change) between primary vs metastatic SKCM-TCGA. Genes with an at least 2-fold higher expression in metastatic SKCM are shown in red (*n*=16), genes with lower expression in metastases are shown in blue (*n*=22).

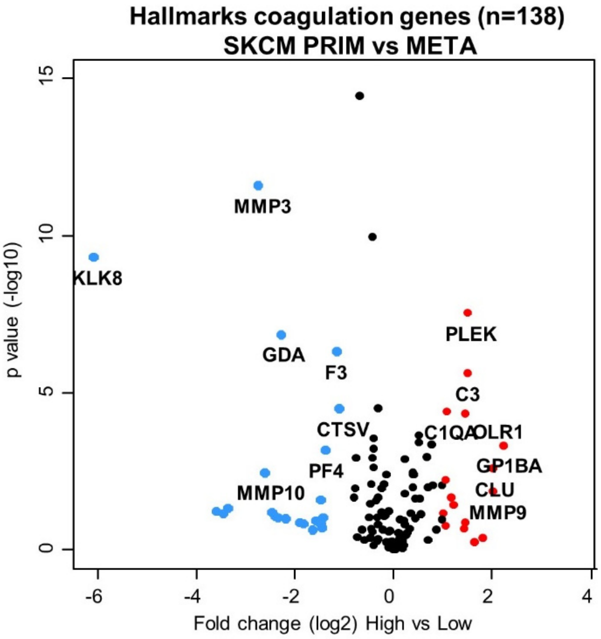

Figure S2. Analysis of the TME of SKCM metastases stratified according to the *GP1BA* expression. A. Stromal / immune score analysis of metastatic SKCM, stratified according to their expression of *GP1BA*. B. Immune cell composition of tumors analysed by CIBERSORTx analysis (relative fraction / absolute score) in metastatic SKCM from TCGA, stratified according to their expression of *GP1BA*. * p<0.05, ** p<0.01, *** p<0.001

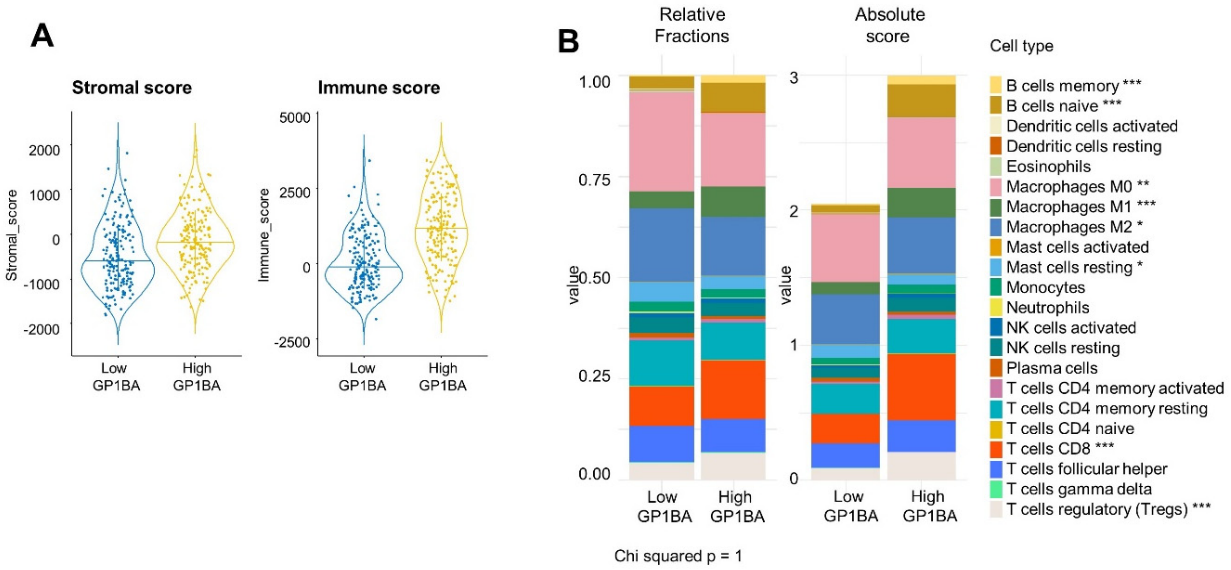

Figure S3. CRG expression according to metastatic sites in SKCM from TCGA. Heatmap showing the expression of the 138 CRG in different metastatic sites in SKCM from TCGA, showing median Z scores values for each site. Red=high expression, blue=low expression.

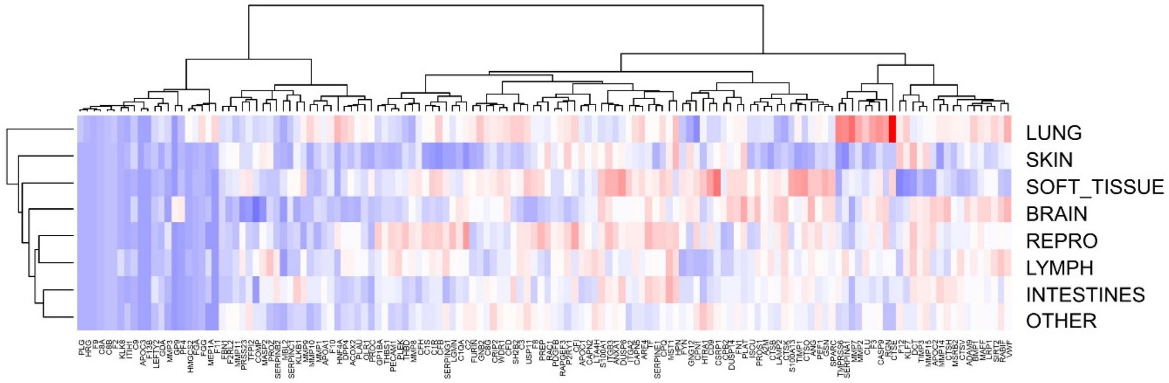

Figure S4. Regulation of *F3* expression in lung metastases of SKCM. A. Comparison of Copy Number Alterations (CNA) in lung metastases of SKCM with low vs high *F3* (*n*=8). B. *F3* methylation levels in lung metastases of SKCM stratified according to *F3* expression.

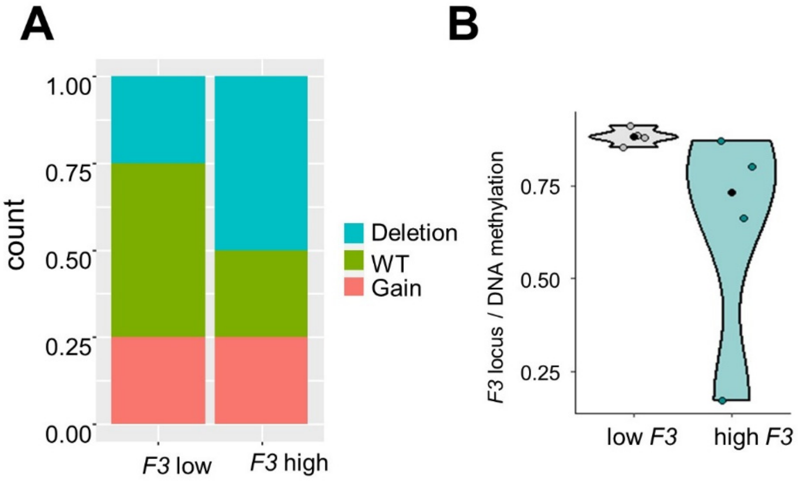

Figure S5. Analysis of the TME of lung metastases of SKCM according to F3 expression. A. Stromal score analysis of lung metastases of SKCM, stratified according to their expression of *F3*. B. Pearson correlation plot of *F3* expression with *FAP* for SKCM lung metastases. C. Immune score analysis of lung metastases of SKCM, stratified according to their expression of *F3*. D. Immune cell composition of tumors analysed by CIBERSORTx analysis (relative fraction / absolute score) in individual lung metastases of SKCM from TCGA, ranked according to their expression of *F3*.

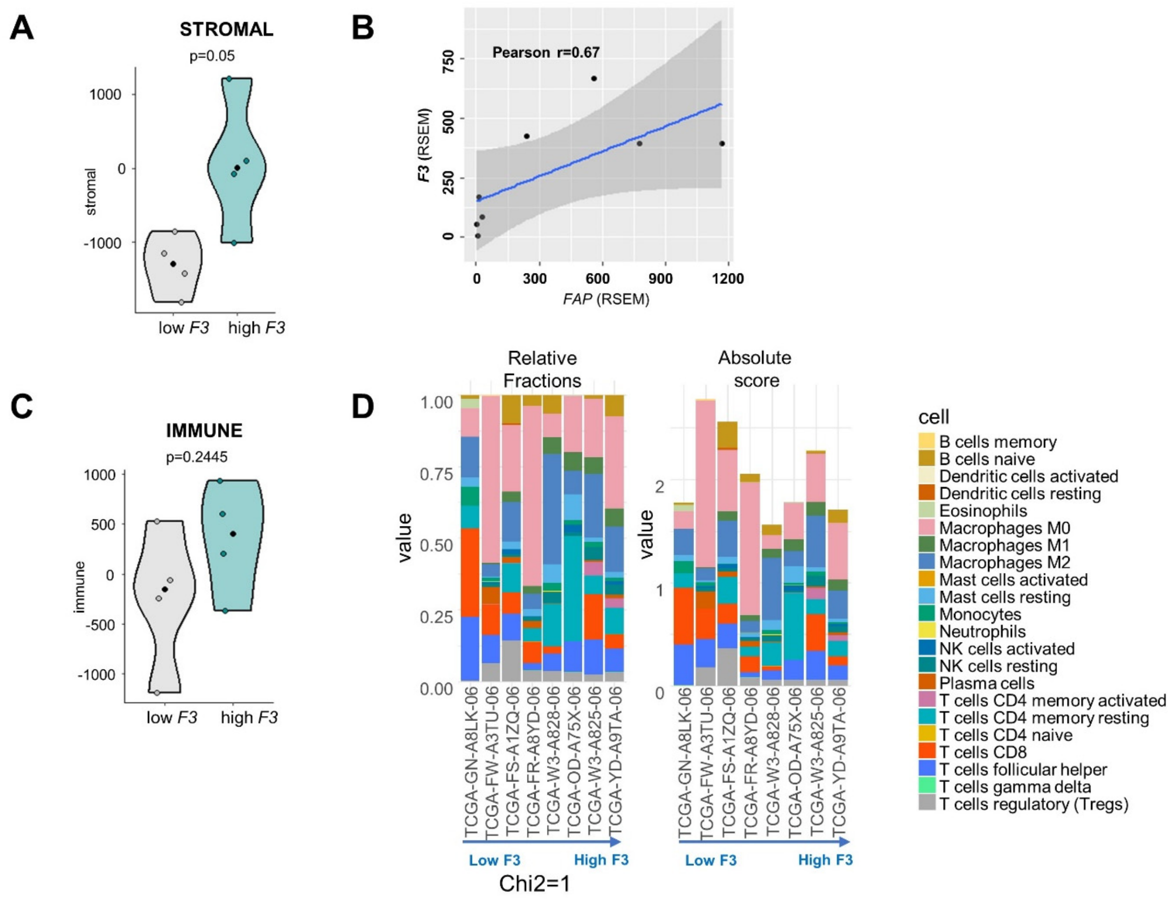

Figure S6: Inter- and intra-tumor variance in CRG expression in metastases of SKCM. A. Heterogeneity in expression of *F3*, *PLAT*, *SERPINE1* in cancer cells from individual patients from GSE72056 (RNA-seq, TPM log2, *n*=1257 cells). B. Dotplot showing the inter- and intra-tumor variance for each CRG, showing the existence of a general positive correlation between inter- and intra-tumor variance in CRG expression.

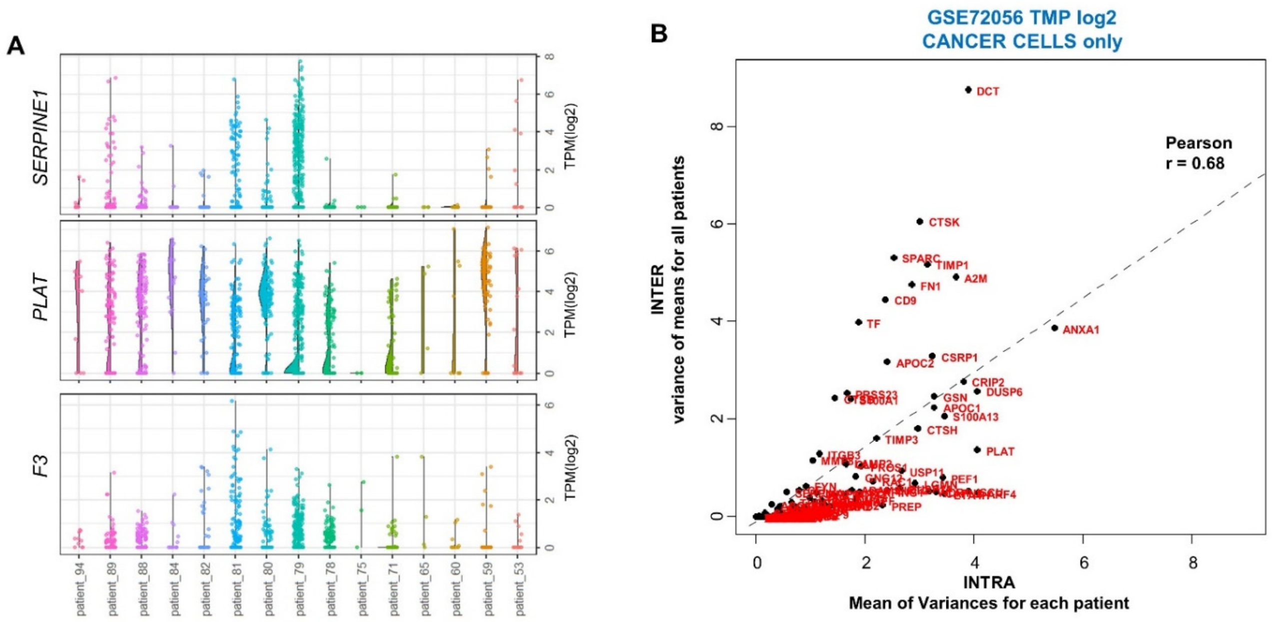

Figure S7. Tumor cell-type specific gene expression analysis of complement genes.A. Cell type specific gene expression analysis of three genes of the complement, *C1R*, *C1S* and *C3*, examined in single cells from 19 metastases (*n*=4645 cells) of SKCM (GSE72056), showing high expression in CAF. B. Venn diagram showing the overlap in expression of the three complement genes. A gene enrichment analysis addressing the context of expression with a GO term analysis based on the genes most correlated with the expression of *C3*.

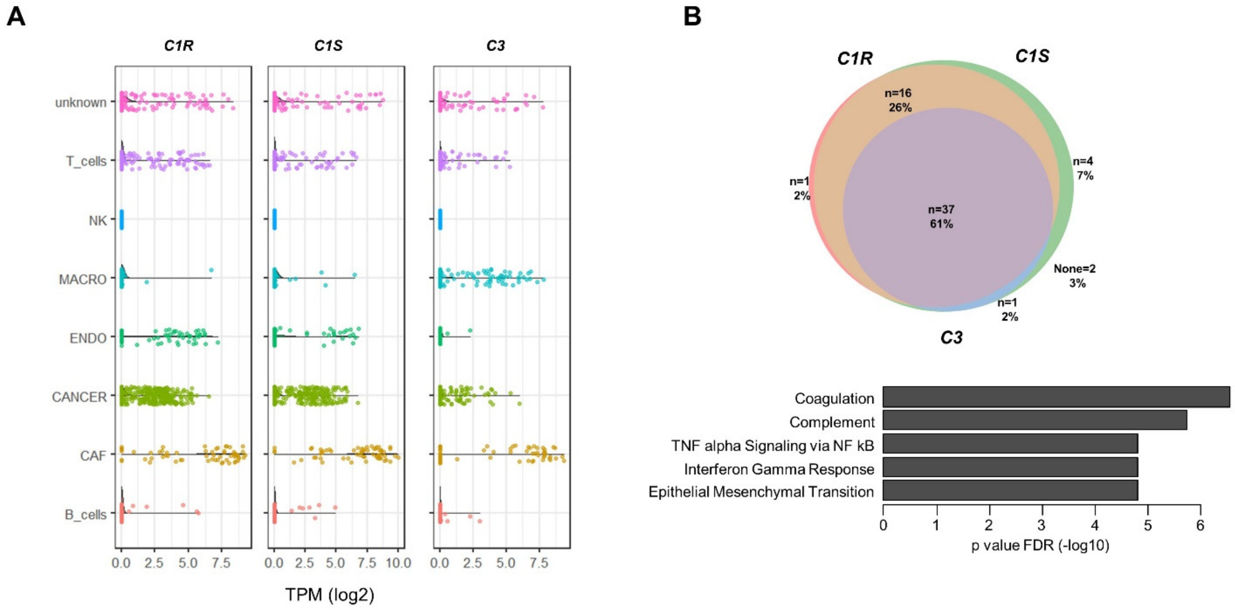

Figure S8. Combined UMAP analysis of CRG expression in single cells from GSE72056 (SKCM metastases) and GSE255299 (CTC). The analysis is based on gene expression data for 138 CRG (TPM log2).

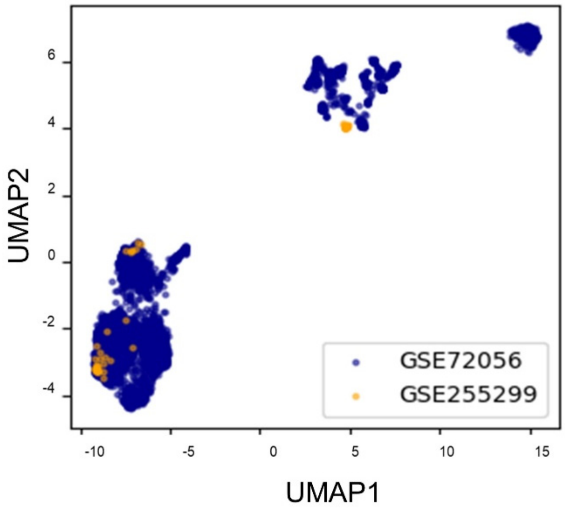

Figure S9. *F3*, *SERPINE1* and *PLAT* expression analysis in single-cancer cells from SKCM metastases *vs* CTC.

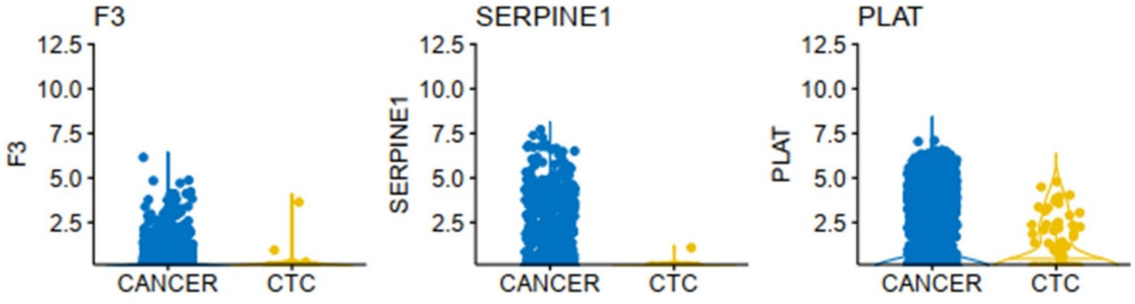

****
