# Supplementary material for: The Dynamic Landscape of the Coagulome of Metastatic Malignant Melanoma
Source: Int J Mol Sci. 2025 Feb 8;26(4):1435. doi: 10.3390/ijms26041435 (PMC11855523; doi:10.3390/ijms26041435)
Supplement: Supplementary file 1 [file ijms-26-01435-s001.zip › Supplementary Materials and Methods.pdf]

Supplementary Materials and Methods to J-P. Arnault et al.

The dynamic landscape of the coagulome of metastatic malignant melanoma

The Cancer Genome Atlas (TCGA) cohorts

Data from the TCGA-SKCM (SKin Cutaneous Melanoma, Firehose Legacy) cohort were retrieved from https://cbioportal.org [49,50] on the 5th of January 2024. TCGA-SKCM consists of 480 samples, 472 of which have gene expression data and were maintained for further analysis (n=472 samples, including 103 primary samples and 369 metastatic samples). 71 of the metastatic TCGA-SKCM have annotation regarding the metastatic location. We retrieved basic clinical, pathological, transcriptomic data (RNA SeqV2data normalised using RNA-Seq by Expectation Maximization: RSEM), DNA methylation (*n*=472) and gene copy number alterations (*n*=367) (GISTIC 2.0: Genomic Identification of Significant Targets in Cancer). The TCGA-SKCM cohort was composed of 255 Stage I/II, 185 stage III and 32 stage IV tumors (American Joint Commission on Cancer, AJCC 7th edition). The average age was 58 years and 94% of patients were Caucasian.

RNA-seq data from the following other TCGA tumor cohorts were retrieved (cBioportal, Firehose Legacy, RSEM, 5th of January 2024): bladder urothelial carcinoma (BLCA, *n*=408), brain lower grade glioma (LGG, *n*=530), breast invasive carcinoma (BRCA, *n*=1100), cervical

squamous cell carcinoma (CESC, *n*=306), colorectal adenocarcinoma (COAD, *n*=382), esophageal adenocarcinoma (ESCA, *n*=185), glioblastoma multiforme (GBM, *n*=166), head and neck squamous cell carcinoma (HNSC, *n*=522), kidney renalclear cell carcinoma (KIRC, *n*=534), kidney renal papillary cell carcinoma (KIRP, *n*=291), lung adenocarcinoma (LUAD, *n*=517), lung squamous cell carcinoma (LUSC, *n*=501), ovarian serous cystadenocarcinoma (OV, *n*=307), pancreatic adenocarcinoma (PAAD, *n*=179), prostate adenocarcinoma (PRAD, *n*=498), sarcoma (SARC, *n*=263), stomach adenocarcinoma (STAD, *n*=415), thyroid carcinoma (THCA, *n*=509), uterine corpus endometrial carcinoma (UCEC, *n*=177).

Other Melanoma Cohorts

Transcriptomic data from the Pan-cancer Analysis of Advanced and Metastatic Tumors cohort from BCSGC (British Colombia Genome Sciences Centre) [34], a pan-cancer collection of advanced and metastatic tumors, were retrieved on the 5th of January 2024 through cBioportal (RPKM). Of the 438 metastatic samples from various primary tumors, we retrieved 16 melanoma metastases, 127 breast cancer metastases, 27 Non-small cell lung carcinoma (NSCLC) metastases, 22 sarcoma metastases. The metastatic sites for the melanoma tumors analysed were : liver (*n*=7), lung (*n*=2), lymph node (*n*=4), skin (*n*=2) and bone (*n*=1). Of the 16 patients, seven were treated with immune checkpoint blockers (ICB).

Transcriptomic data from metastatic melanoma samples from the Posthumous Evaluation of Advanced Cancer environment (PEACE) study [25] were retrieved on the 1st of February 2024. Among the 14 patients with sequencing data available, 4 had metastatic SKCM with more than one metastatic lung sample (*n*=36 samples analyzed). All four patients analysed were treated with ICB (3 progressive disease and 1 partial response).

Single cell melanoma analyses

Single cell transcriptomic data from melanoma samples were retrieved from GSE72056 [29] and GSE255299 [31] via the Gene Expression Omnibus (GEO) in January 2024.

In GSE72056, freshly resected samples were obtained from 19 patients with a range of clinical and therapeutic backgrounds (average age 68 years), of which 10 were lymphoid metastases (nodal and spleen), and 8 distant metastases. Tumor samples were disaggregated, sorted into single cells and profiled by single-cell RNA-seq. GSE72056 includes data for *n*= 4645 single cells (TMP log2).

Single cell transcriptomic data were available for *n*=75 circulating tumor cells (CTC) from seven patients with SKCM in GSE255299 [31]. The corresponding 7 patients (2 stage II and 5 stage III tumors; AJCC 7th ed.) were treated with ICB. The average age was 54 years. Raw gene expression data was normalised according to the data processing protocol used by Tirosh *et al.* (2016) [29]. Raw counts were divided by transcript length and normalised for a total of 100,000 per sample.

Gene Set Enrichment Analysis (GSEA), Gene ontology (GO) analysis

Gene Set Enrichment Analysis (GSEA) was performed using the Java GSEA desktop application, (https://www.gsea-msigdb.org/gsea/index.jsp). Curated Hallmark gene sets downloaded from the GSEA website were used to compute their overrepresentation. The analyses were done using 1,000 permutations. Leading edge analysis for the Hallmarks “Coagulation“ gene set was done to identify the most contributive genes.

Gene Ontology (GO) analyses were carried out using PANTHER (Protein ANalysis THrough Evolutionary Relationships, http://www.pantherdb.org/) [52]. A statistical over-representation

test/GO Biological Process complete analysis was performed using Fisher's exact test with FDR correction. The comparisons were performed against the Homo sapiens all genes database. Enrichr was used to identify pathway or ontology enrichment in specific gene lists [51].

Tumor microenvironment analysis

STROMAL and IMMUNE scores were used to evaluate tumor purity, i.e. the infiltration levels of stromal and immune cells based on gene expression data http://bioinformatics.mdanderson.org/estimate/ [53]. The CIBERSORTx algorithm (https://cibersortx.stanford.edu), accessed on the 5 April 2024, was used to quantify the levels of 22 cell subsets using the validated leukocyte gene signature matrix LM22 (absolute and fractions) [54,55].
